# Cyclooxygenase-2/sclerostin mediates TGF-β1-induced calcification in vascular smooth muscle cells and rats undergoing renal failure

**DOI:** 10.18632/aging.103827

**Published:** 2020-11-06

**Authors:** Fang He, Ling Li, Pei-Pei Li, Yan Deng, Yuan-Yuan Yang, Yi-Xuan Deng, Hong-Hong Luo, Xin-Tong Yao, Yu-Xi Su, Hua Gan, Bai-Cheng He

**Affiliations:** 1 Department of Pharmacology, School of Pharmacy, Chongqing Medical University, Chongqing 400016, China; 2 Department of Nephrology, The First Affiliated Hospital of Chongqing Medical University, Chongqing 400016, China; 3 Chongqing Key Laboratory of Biochemistry and Molecular Pharmacology, Chongqing Medical University, Chongqing 400016, China; 4 Department of Orthopedic, Children Hospital of Chongqing Medical University, Chongqing 400014, China

**Keywords:** vascular calcification, TGF-β1, COX-2, sclerostin, chronic kidney disease

## Abstract

In this study, we studied the effect and possible mechanism of TGF-β1 on vascular calcification. We found that the serum levels of TGF-β1 and cycloxygenase-2 (COX-2) were significantly increased in patients with chronic kidney disease. Phosphate up regulated TGF-β1 in vascular smooth muscle cells (VSMCs). TGF-β1 decreased the markers of VSMCs, but increased osteogenic markers and calcification in aortic segments. The phosphate-induced osteogenic markers were reduced by the TGFβR I inhibitor (LY364947), which also attenuated the potential of phosphate to reduce VSMC markers in VSMCs. Both phosphate and TGF-β1 increased the protein level of β-catenin, which was partially mitigated by LY364947. TGF-β1 decreased sclerostin, and exogenous sclerostin decreased the mineralization induced by TGF-β1. LY364947 reduced the phosphate and TGF-β1 induced COX-2. Meanwhile, the effects of TGF-β1 on osteogenic markers, β-catenin, and sclerostin, were partially reversed by the COX-2 inhibitor. Mechanistically, we found that p-Smad2/3 and p-CREB were both enriched at the promoter regions of sclerostin and β-catenin. TGF-β1 and COX-2 were significantly elevated in serum and aorta of rats undergoing renal failure. Therapeutic administration of meloxicam effectively ameliorated the renal lesion. Our results suggested that COX-2 may mediate the effect of TGF-β1 on vascular calcification through down-regulating sclerostin in VMSCs.

## INTRODUCTION

Chronic kidney disease (CKD) is a risk factor for cardiovascular diseases, such as vascular calcification. Although a high level of calcium or phosphate is associated with vascular calcification, the underlying mechanism remains unclear. Vascular calcification shares similar characteristics with osteogenic differentiation. Various factors or signals, such as transforming growth factor-beta (TGF-β), bone morphogenetic protein (BMP), Wnt/β-catenin, and IL-1β, can regulate the osteogenic differentiation process [1–3]. Thus, these factors may also be implicated in vascular calcification.

TGF-β regulates diverse physiological processes, such as cell proliferation, differentiation, apoptosis, and migration. It includes three isoforms at least [4, 5]. TGF-β1 may regulate osteogenic differentiation in multiple mesenchymal progenitor cells. Its role remains controversial because TGF-β1 may promote or inhibit osteogenic differentiation depending on the cell types, cellular context, or concentrations [6, 7]. Besides osteogenic differentiation, TGF-β1 also regulates vascular smooth muscle cell calcification and valve calcification in the heart [2, 8]. However, the detailed mechanism underlying the effect of TGF-β1 on calcification remains unclear.

Wnt/β-catenin plays an essential role in skeletal development [9]. It was reported that abnormal vascular calcification is mediated by Wnt/β-catenin signaling [3], and inhibition of Wnt/β-catenin seemingly protected against phosphate-induced calcification in VSMCs [10]. It is conceivable that TGF-β1 may crosstalk with Wnt/β-catenin to regulate calcification in VSMCs. Cyclooxygenase (COX) is a critical enzyme in the biological synthesis of prostaglandins. Its inhibitors, known as non-sterol anti-inflammatory drugs (NSAIDs), have been widely used as antipyretics, analgesics, and anti-inflammatory drugs. COX-2 expression can be induced by various factors, such as LPS, IL-1, TNF-α, and hypoxia [11–14]. COX-2-specific inhibitors have been widely used as analgesics because of their very few gastric adverse effects. COX-2 also regulates bone metabolism. Knockout or inhibition of *Cox-2* delayed bone fracture healing, and the silence of *Cox-2* inhibited the osteogenic differentiation induced by BMP9 [15, 16]. COX-2-specific inhibitors, such as rofecoxib and meloxicam, reduced ectopic ossification after hip arthroplasty operation or spine corn injury [17, 18]. These pieces of evidence indicated that COX-2-specific inhibitors might possess the potential to reduce the abnormal ossification and ameliorate vascular calcification in CKD. Although the administration of aspirin did not reduce the endpoint of patients with CKD, the outcome may be different for the usage of COX-2-specific inhibitors [19, 20].

In this study, we investigated the possible role of TGF-β1 in vascular calcification, analyzed the crosstalk between TGF-β1 and COX-2 or Wnt/β-catenin, and evaluated the effects of meloxicam on rats undergoing renal failure.

## RESULTS

### TGF-β1 induces calcification in VSMCs and aortic segments

**Figure 1:**
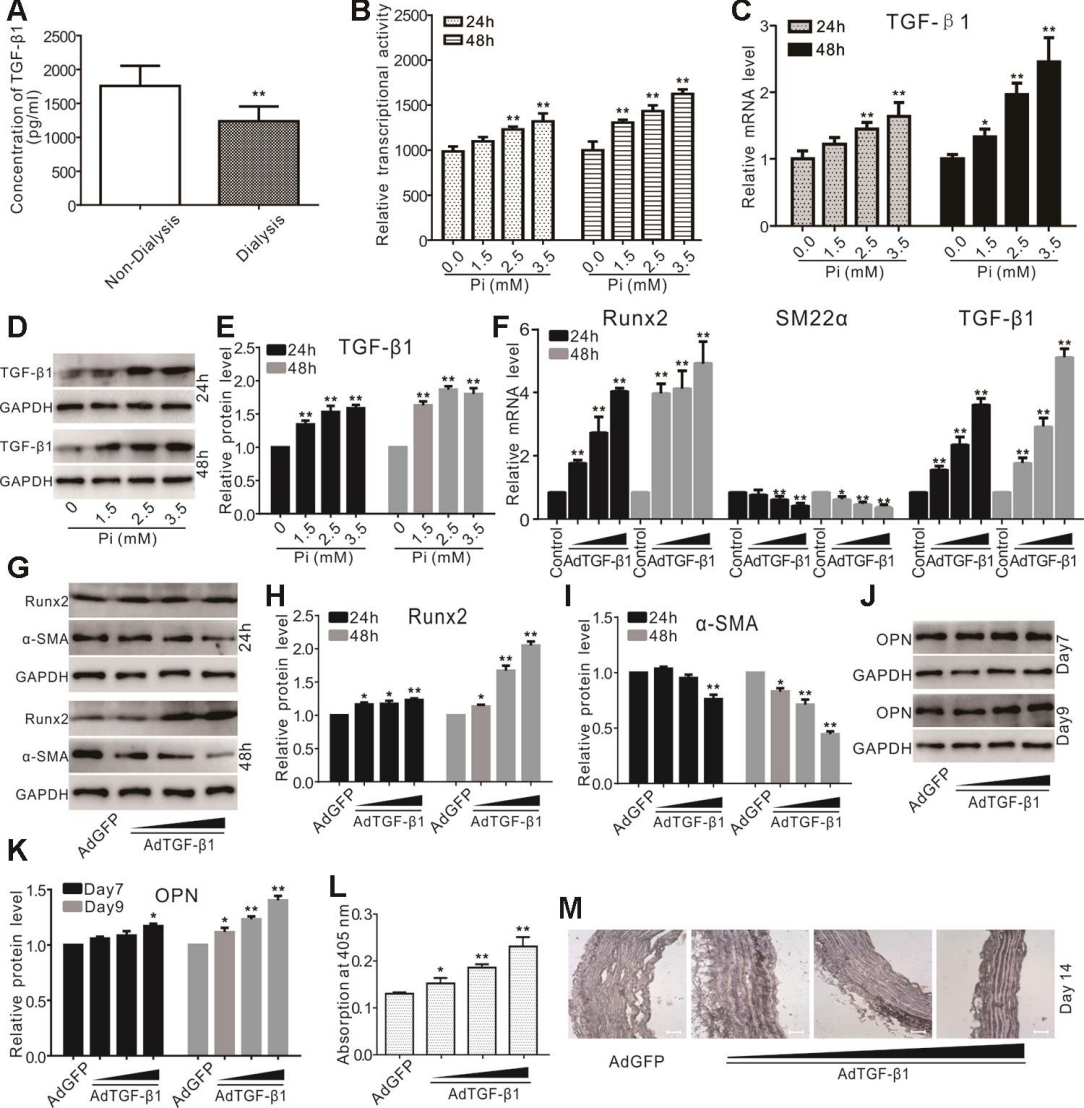
**Effects of TGF-β1 on calcification in VSMCs and aortic segments.** (**A**) ELISA assay results show the serum levels of TGF-β1 in patients with CKD experienced with dialysis or non-dialysis (n=40) ("**" p<0.01 vs. non-dialysis). (**B**) Reporter assay results show the effect of high phosphate on TGF-β signaling ("**" p<0.01 vs. control). (**C**) Real-time PCR assay results show the impact of high phosphate on mRNA expression of TGF-β1 ("*" p<0.05 vs. control, "**" p<0.01 vs. control). (**D**) Western blot assay results show the effect of high phosphate on TGF-β1; GAPDH was used as a loading control. (**E**) Quantification results of Western blot assay show the effect of high phosphate on TGF-β1 ("**" p<0.01 vs. control). (**F**) Real-time PCR assay results show the effect of TGF-β1 recombinant adenovirus on mRNA expression of *Runx2, SM22α,* and TGF-β1 ("*" p<0.05 vs. control, "**" p<0.01 vs. control). (**G**) Western blot assay results show the effect of TGF-β1 on Runx2 and α-SMA in VSMCs; GAPDH was used as a loading control. (**H**) Quantification results of Western blot assay show the effect of TGF-β1 on Runx2 in VSMCs("*" p<0.05 vs. control, "**" p<0.01 vs. control). (**I**) Quantification results of Western blot assay show the effect of TGF-β1 on α-SMA in VSMCs ("*" p<0.05 vs. control, "**" p<0.01 vs. control). (**J**) Western blot assay results show the effect of TGF-β1 on OPN in VSMCs; GAPDH was used as a loading control. (**K**) Quantification results of Western blot assay show the effect of TGF-β1 on OPN in VSMCs ("*" p<0.05 vs. control, "**" p<0.01 vs. control). (**L**) Quantitative analysis results of Alizarin Red S staining show the effect of exogenous TGF-β1 on inducing mineralization in VSMCs ("*" p<0.05 and "**" p<0.01 vs. control). (**M**) The Von Kossa staining results show the effect of TGF-β1 on calcification in thoracic segments (Scale=100 μM), Pi: phosphate.

TGF-β1 is a pleiotropic cytokine that is associated with calcification. Using the serum samples from patients with CKD, we found that the serum levels of TGF-β1 in the patients with CKD without dialysis were much higher than those of patients with CKD with regular dialysis (Figure 1A). In primary VSMCs, we showed that phosphate effectively increased TGF-β reporter activity (Figure 1B). The real-time PCR analysis revealed that phosphate up regulated the mRNA of TGF-β1 in a concentration-dependent fashion in VSMCs, which was also confirmed by Western blot analysis (Figure 1C–1E). These results suggest that TGF-β1 may participate in the phosphate-induced vascular calcification.

We further conducted real-time PCR analysis and found that TGF-β1 increased the mRNA level of Runx2, but decreased that of SM22α in VSMCs (Figure 1F). Similar results were obtained from western blot assay (Figure 1G–1I). TGF-β1 effectively increased the protein level of osteopontin (OPN) (Figure 1J–1K) and enhanced the matrix mineralization in VSMCs (Figure 1L). In the organ culture assay, we demonstrated that TGF-β1 effectively induced calcification in thoracic artery segments (Figure 1M). As OPN and matrix mineralization have been used as osteogenic or calcification markers, these results strongly suggest that TGF-β1 may be involved in promoting calcification in VSMCs.

### TGF-β1 potentiates phosphate-induced calcification markers in VSMC cells

**Figure 2:**
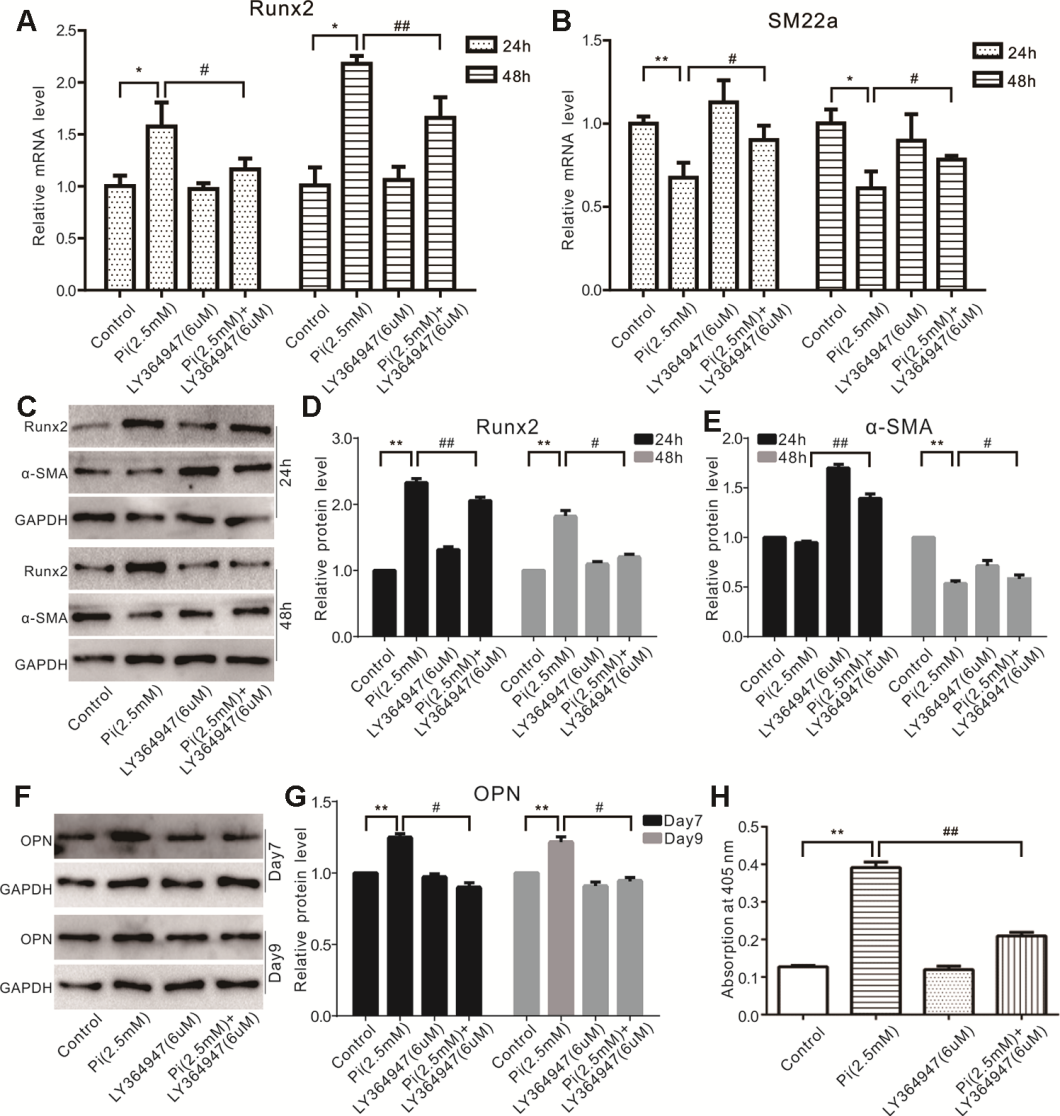
**Effects of TGF-β1 on high phosphate-induced calcification markers in VSMCs cells.** (**A**) Real-time PCR assay results show the effect of high phosphate and/or LY364947 on mRNA of *Runx2* in VSMCs cells ("*" p<0.05 vs. control; "#" p<0.05 and "##” p<0.01 vs. high phosphate group). (**B**) Real-time PCR assay results show the effect of high phosphate and/or LY364947 on mRNA expression of *SM22α* in VSMCs cells ("*" p<0.05 and "**" p<0.01 vs. control; "#" p<0.05 vs. high phosphate group). (**C**) Western blot assay results show the effect of high phosphate and/or LY364947 on Runx2 and α-SMA in VSMCs cells; GAPDH was used as a loading control. (**D**) Quantification results of Western blot assay show the effect of high phosphate and/or LY364947 on Runx2 in VSMCs cells ("**" p<0.01 vs. control; "#" p<0.05 and "##" p<0.01 vs. high phosphate group). (**E**) Quantification results of Western blot assay show the effect of high phosphate and/or LY364947 on α-SMA in VSMCs cells ("**" p<0.01 vs. control; "#" p<0.05 and "##" p<0.01 vs. high phosphate group). (**F**) Western blot assay results show the effect of high phosphate and/or LY364947 on OPN in VSMCs cells; GAPDH was used as a loading control. (**G**) Quantification results of Western blot assay show the effect of high phosphate and/or LY364947 on OPN in VSMCs cells ("**" p<0.01 vs. control; "#" p<0.05 vs. high phosphate group). (**H**) Quantitative analysis results of Alizarin Red S staining show the effect of TGF-β1 on mineralization in VSMCs ("**" p<0.01 vs. control; "##" p<0.01 vs. high phosphate group). Pi: phosphate, LY364947: TGF-β type I receptor (TGFβRI) specific inhibitor.

To test whether TGF-β1 played any role in phosphate-induced calcification, we conducted real-time PCR analysis and found that phosphate effectively up regulated Runx2 expression. Although it did not exert any effect, the TGF-β type I receptor (TGF-βR) inhibitor LY364947 was shown to suppress phosphate-induced Runx2 expression (Figure 2A) significantly. Conversely, phosphate was shown to decrease SM22α expression in VSMCs, which was partially reversed by LY364947 (Figure 2B). Furthermore, phosphate up regulated the protein level of Runx2 and decreased that of α-SMA, which could be attenuated by LY364947 (Figure 2C–2E). As expected, phosphate increased the protein level of OPN and promoted mineralization in VSMCs cells, which could also be suppressed by LY364947 (Figure 2F–2H). These results suggest that TGF-β1 may at least in part play a role in mediating phosphate-induced calcification in VSMCs.

### Both phosphate and TGF-β1 enhance Wnt/β-catenin signaling in VSMCs

**Figure 3:**
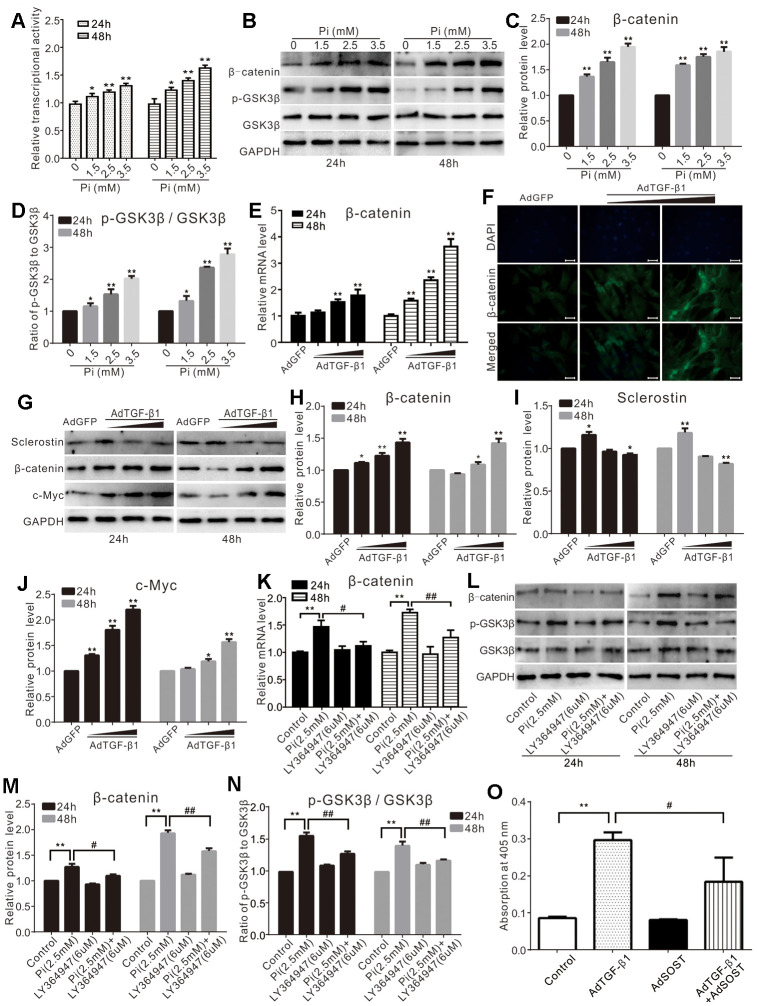
**Effects of high phosphate and/or TGF-β1 on Wnt/β-catenin in VSMCs.** (**A**) Luciferase reporter assay results show the effect of high phosphate on Wnt/β-catenin signal ("*" p<0.05 and "**" p<0.01 vs. control). (**B**) Western blot assay results show the effect of high phosphate on β-catenin, p-GSK-3β, and GSK-3β in VSMCs cells; GAPDH was used as a loading control. (**C**) Quantification results of Western blot assay show the effect of high phosphate on β-catenin in VSMCs cells ("**" p<0.01 vs. control). (**D**) Quantification results of Western blot assay show the effect of high phosphate on p-GSK-3β and GSK-3β in VSMCs cells ("*" p<0.05 and "**" p<0.01 vs. control). (**E**) Real-time PCR assay results show the effect of TGF-β1 on mRNA expression of β-catenin in VSMCs cells ("**" p<0.01 vs. control). (**F**) Immunofluorescent staining results show the effect of TGF-β1 on β-catenin and its localization in VSMCs (Scale=50 μM). (**G**) Western blot assay results show the effect of TGF-β1 on sclerostin, β-catenin, and c-Myc in VSMCs cells, GAPDH was used as a loading control. (**H**) Quantitative results of Western blot assay show the effect of TGF-β1 on β-catenin in VSMCs cells ("*" p<0.05 and "**" p<0.01 vs. control). (**I**) Quantitative results of Western blot assay show the effect of TGF-β1 on sclerostin in VSMCs cells ("*" p<0.05 and "**" p<0.01 vs. control). (**J**) Quantitative results of Western blot assay show the effect of TGF-β1 on c-Myc in VSMCs cells ("*" p<0.05 and "**" p<0.01 vs. control). (**K**) Real-time PCR assay results show the effect of high phosphate and/or LY364947 on mRNA expression of β-catenin in VSMCs cells ("**" p<0.01 vs. control; "#" p<0.05 and "##” p<0.01 vs. high phosphate group). (**L**) Western blot assay results show the effect of high phosphate and/or LY364947 on β-catenin, p-GSK-3β, and GSK-3β in VSMCs cells; GAPDH was used as a loading control. (**M**) Quantitative results of Western blot assay show the effect of high phosphate and/or LY364947 on β-catenin in VSMCs cells ("**" p<0.01 vs. control; "#" p<0.05 and "##” p<0.01 vs. high phosphate group). (**N**) Quantitative results of Western blot assay show the effect of high phosphate and/or LY364947 on p-GSK-3β and GSK-3β in VSMCs cells ("**" p<0.01 vs. control; "##” p<0.01 vs. high phosphate group). (**O**) Quantitative analysis results of Alizarin Red S staining show the effect of TGF-β1 and/or sclerostin on mineralization in VSMCs ("**" p<0.01 vs. control; "#" p<0.05 vs. TGF-β1 group). Pi: phosphate, LY364947: TGFβRI-specific inhibitor.

Wnt/β-catenin signaling regulates cell proliferation and differentiation, as well as calcification. We found that phosphate increased the Tcf/Lef reporter activity in a concentration-dependent manner in VSMCs (Figure 3A). Phosphate was shown to increase the protein level of β-catenin and p-GSK3β, without affecting the total GSK3β (Figure 3B–3D). TGF-β1 was shown to increase the mRNA and protein level of β-catenin in VSMCs cells (Figure 3E, 3F). Upon the same time, TGF-β1 increased the protein level of β-catenin and c-Myc while reducing the level of sclerostin in VSMCs (Figure 3G–3J). Meanwhile, we also found that phosphate significantly up-regulated β-catenin at both mRNA and protein levels in VSMCs, and increased the level of p-GSK3β; but these effects were effectively blocked by LY364947 (Figure 3K–3N). Over-expression of sclerostin (SOST) was shown to inhibit TGF-β1-induced mineralization effectively (Figure 3O). These results suggest that activation of Wnt/β-catenin signaling may be necessary for calcification induced by phosphate and/or TGF-β1.

### COX-2 is associated with phosphate and TGF-β1 induced calcification in VSMCs

**Figure 4:**
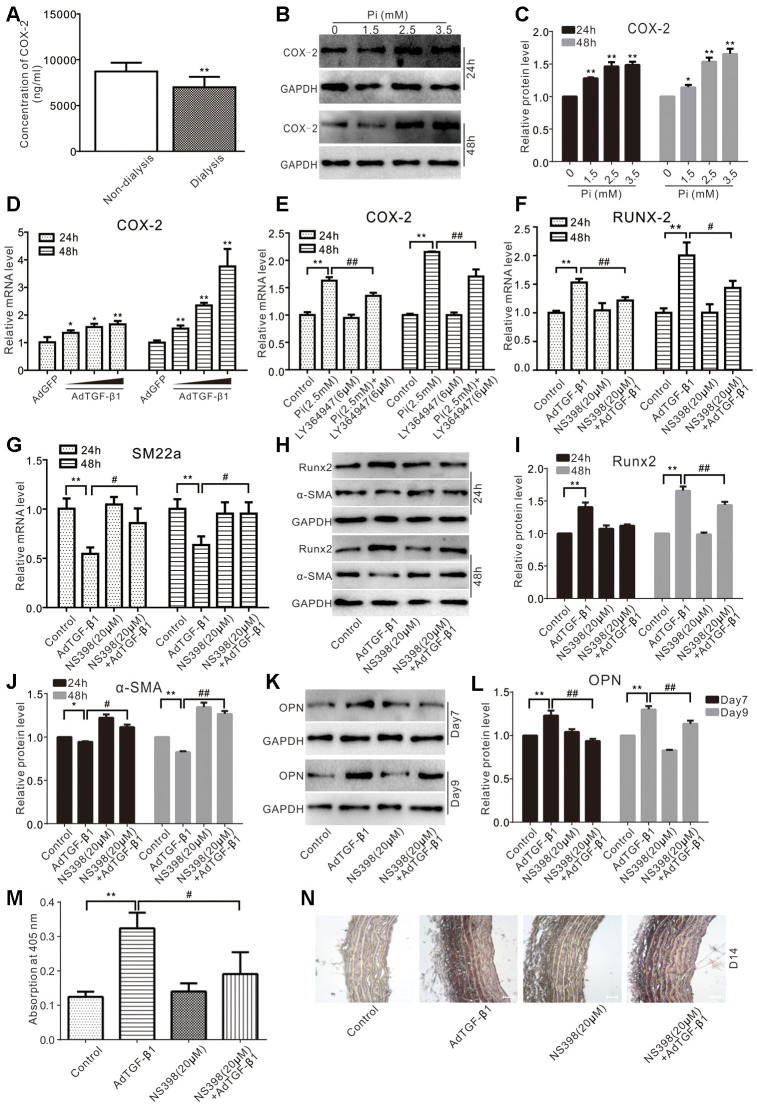
**Effects of COX-2 on high phosphate and/or TGF-β1 induced calcification in VSMCs.** (**A**) ELISA analysis results show the serum level of COX-2 in CKD patients treated with regular dialysis or non-dialysis (n=48) ("**" p<0.01 vs. non-dialysis). (**B**) Western blot assay results show the effect of high phosphate on COX-2 in VSMCs cells; GAPDH was used as a loading control. (**C**) Quantitative results of Western blot assay show the effect of high phosphate on COX-2 in VSMCs cells ("*" p<0.05 and "**" p<0.01 vs. control). (**D**) Real-time PCR assay results show the effect of TGF-β1 on mRNA expression of *Cox-2* in VSMCs cells ("*" p<0.05 and "**" p<0.01 vs. control). (**E**) Real-time PCR assay results show the effect of high phosphate and/or LY364947 on mRNA expression of *Cox-2* in VSMCs cells ("**" p<0.01 vs. control, "##" p<0.01 vs. high phosphate group). (**F**) Real-time PCR assay results show the effect of TGF-β1 and/or NS398 on expression mRNA of *Runx-2* in VSMCs cells ("**" p<0.01 vs. control, "#" p<0.05 and "##” p<0.01 vs. TGF-β1 group). (**G**) Real-time PCR assay results show the effect of TGF-β1 and/or NS398 on mRNA expression of *SM22α* in VSMCs cells ("**" p<0.01 vs. control, "#" p<0.05 vs. TGF-β1 group). (**H**) Western blot assay results show the effect of TGF-β1 and/or NS398 on Runx2 and α-SMA in VSMCs cells, GAPDH was used as a loading control. (**I**) Quantitative results of Western blot assay show the effect of TGF-β1 and/or NS398 on Runx2 in VSMCs cells ("**" p<0.01 vs. control, "##" p<0.01 vs. TGF-β1 group). (**J**) Quantitative results of Western blot assay show the effect of TGF-β1 and/or NS398 on α-SMA in VSMCs cells ("*" p<0.05 and "**" p<0.01 vs. control, "#" p<0.05 and "##" p<0.01 vs. TGF-β1 group). (**K**) Western blot assay results show the effect of TGF-β1 and/or NS398 on OPN in VSMCs cells; GAPDH was used as a loading control. (**L**) Quantitative results of Western blot assay show the effect of TGF-β1 and/or NS398 on OPN in VSMCs cells ("**" p<0.01 vs. control, "##" p<0.01 vs. TGF-β1 group). (**M**) Quantitative analysis results of Alizarin Red S staining show the effect of TGF-β1 and/or NS398 on mineralization in VSMCs ("**" p<0.01 vs. control; "#" p<0.05 vs. TGF-β1 group). (**N**) The Von Kossa staining results show the effect of TGF-β1 and/or NS398 on calcification in aortic segments (Scale=100 μM). Pi: phosphate, LY364947: TGFβRI-specific inhibitor, NS398: COX-2-specific inhibitor.

We analyzed a panel of serum samples of patients with CKD, and we found that the serum level of COX-2 in the patients with CKD receiving regular dialysis was much lower than that of those receiving no dialysis (Figure 4A), suggesting that COX-2 may be associated with the disease process of CKD. We also showed that phosphate increased the protein level of COX-2 in VSMCs (Figure 4B, 4C), and TGF-β1 increased the mRNA level of COX-2 (Figure 4D). But, the potential of phosphate on up-regulating COX-2 was significantly repressed by LY364947 (Figure 4E). Furthermore, TGF-β1 was shown to up regulate the mRNA level of Runx2, which could be effectively blocked by NS398 (Figure 4F). Conversely, TGF-β1 decreased the mRNA level of SM22α, which could be effectively rescued by NS398 (Figure 4G). Accordingly, TGF-β1 was shown to increase the protein level of Runx2 while reducing that of α-SMA, which could be partially reversed by NS398 (Figure 4H–4J). Similarly, TGF-β1 increased the protein level of OPN and mineralization in VSMCs, both of which were inhibited by NS398 (Figure 4K–4M). We also conducted organ culture assays and found that TGF-β1 increased calcification in aortic segments, which was partially inhibited by NS398 (Figure 4N). Thus, these results strongly suggest that COX-2 may mediate phosphate and/or TGF-β1-induced calcification in VSMCs.

### TGF-β1 and COX-2 activate Wnt/β-catenin signaling in VSMCs

**Figure 5:**
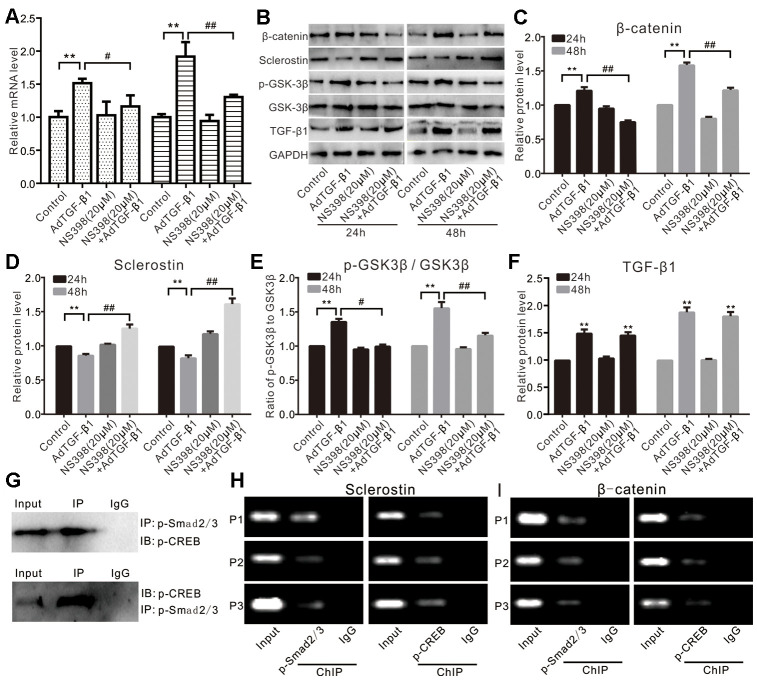
**Effects of TGF-β1 and/or COX-2 on Wnt/β-catenin in VSMCs.** (**A**) Real-time PCR assay results show the effect of TGF-β1 and/or NS398 on mRNA expression of β-catenin in VSMCs cells ("**" p<0.01 vs. control, "#" p<0.05 and "##” p<0.01 vs. TGF-β1 group). (**B**) Western blot assay results show the effect of TGF-β1 and/or NS398 on β-catenin, sclerostin, p-GSK-3β, and GSK-3β in VSMCs cells; GAPDH was used as a loading control. (**C**) Quantitative results of Western blot assay show the effect of TGF-β1 and/or NS398 on β-catenin in VSMCs cells ("**" p<0.01 vs. control, "##” p<0.01 vs. TGF-β1 group). (**D**) Quantitative results of Western blot assay show the effect of TGF-β1 and/or NS398 on sclerostin in VSMCs cells ("**" p<0.01 vs. control, "##” p<0.01 vs. TGF-β1 group). (**E**) Quantitative results of Western blot assay show the effect of TGF-β1 and/or NS398 on p-GSK-3β and GSK-3β in VSMCs cells ("**" p<0.01 vs. control, "#” p<0.05 and "##” p<0.01 vs. TGF-β1 group). (**F**) Quantitative results of Western blot assay show the effect of AdTGF-β1 and/or NS398 on TGF-β1 in VSMCs cells ("**" p<0.01 vs. control). (**G**) IP assay results show the interaction between p-Smad2/3 and p-CREB in VSMCs. (**H**) ChIP assay results show the recruitment of p-Smad2/3 or p-CREB at the promoter region of sclerostin in VSMCs. (**I**) ChIP assay results show the recruitment of p-Smad2/3 or p-CREB at the promoter region of β-catenin in VSMCs. NS398: COX-2-specific inhibitor.

Next, we analyzed the effect of TGF-β1 or COX-2 on Wnt/β-catenin signaling. Our qPCR assay results showed that TGF-β1 increased the mRNA expression of β-catenin, which was effectively inhibited by NS398 in VSMCs (Figure 5A). Accordingly, we also found that TGF-β1 increased the protein level of β-catenin and p-GSK-3β (without affecting the total GSK-3β) while decreasing the protein level of sclerostin in VSMCs. However, the effect of TGF-β1 on β-catenin, p-GSK-3β, and sclerostin could be effectively reversed by NS398 (Figure 5B–5F). Immunoprecipitation assays indicated that p-Smad2/3 interacts with p-CREB (Figure 5G). Furthermore, we conducted ChIP analysis and found that both p-Smad2/3 and p-CREB were recruited to the promoter regions of sclerostin or β-catenin in VSMCs (Figure 5H and 5I). Therefore, these results suggest that the effect of phosphate or TGF-β1 on Wnt/β-catenin signaling may be at least in part mediated by COX-2 in VSMCs.

### Meloxicam effectively alleviates vascular calcification and renal pathologic lesions in a rat model of adenine-induced renal failure

**Figure 6:**
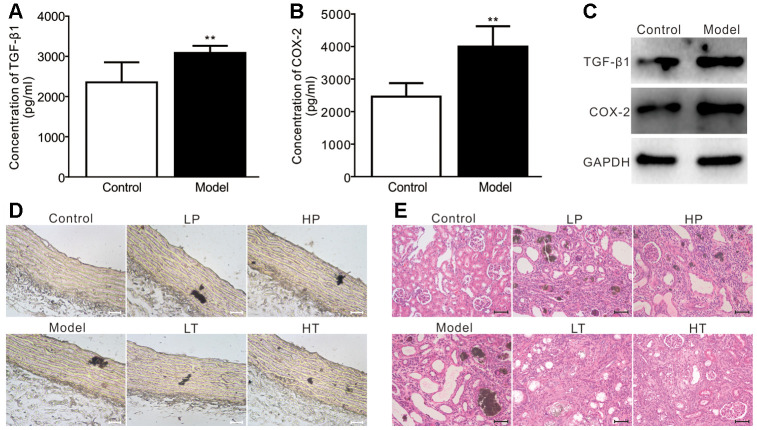
**Effects of meloxicam on adenine-induced renal failure in rats.** (**A**) ELISA analysis results show the serum level of TGF-β1 in adenine-induced rat renal failure (n=10, "**" p<0.01 vs. control). (**B**) ELISA analysis results show the serum level of COX-2 in adenine-induced rat renal failure (n=10, "**" p<0.01 vs. control). (**C**) Western blot assay results show the protein level of TGF-β1 and COX-2 in the aorta from a rat; GAPDH was used as a loading control. (**D**) Von Kossa staining results show the calcification in aorta from adenine-induced rat renal failure, which were treated with preventive or therapeutic administration of meloxicam (Scale=100 μM). (**E**) H&E staining results show the pathological changes in adenine-induced rat renal failure, which were treated with preventive or therapeutic administration of meloxicam (Scale=50 μM). LP: low-dose prevention, HP: high-dose prevention, LT: little dose therapy, HT: high-dose therapy.

Next, we analyzed the effect of the COX-2 inhibitor meloxicam in a rat model of adenine-induced renal failure. Compared with the control group, the serum levels of TGF-β1 and COX-2 were significantly higher in the model group (Figure 6A and 6B). Similarly, the protein levels of TGF-β1 and COX-2 in the aortic arteries of the model group were also higher than those of the control group (Figure 6C). We conducted Von Kossa staining assays and found that adenine-induced calcification was detected in the aortic arteries in the model group. Although the preventive administration of meloxicam reduced vascular calcification, the treatment with meloxicam blocked the calcification more profoundly (Figure 6D). H & E staining revealed that, unlike in the control group, the renal tissue retrieved from the rats in the model group exhibited an apparently disorganized renal structure, generalized sclerosis, increased glomerular size, collapse of some glomerular tufts, and lesions of extraglomerular blood vessels, indicating that the renal failure model had been successfully established.

However, compared with the model group, the renal pathological changes were modestly improved in low-dose prevention (LP) and high-dose prevention (HP) groups. In contrast, the renal lesions were significantly improved in the low-dose therapy (LT) and high-dose therapy (HT) groups (Figure 6E). Namely, the renal structure, glomerular size were similar to those of control groups, fewer glomerular tufts collapse, sclerosis, and extraglomerular blood vessels lesion in the preventive and therapeutic groups.

Compared with the control group, the model group and prevention groups experienced significant body weight loss, which was significantly alleviated in the meloxicam therapy groups (Table 1). Compared with the control group, the serum calcium level increased profoundly in the experimental groups. However, the therapy group with a high dose of meloxicam had significantly lower serum calcium levels than the model group. Similarly, the serum level of phosphate increased markedly in the experimental groups, which was significantly lowered by meloxicam in the prevention and therapy groups compared with the control group (Table 1). Furthermore, we found that the serum levels of BUN and Scr were reduced more profoundly in the therapy group than in the prevention group (Table 1), suggesting that COX-2 inhibitor meloxicam may be used as a therapeutic agent for CKD.

**Table 1:** Effects of meloxicam on body weight and biochemical indexes of the adenine induced renal failure in rats (n=10).

	**Wight (g)**	**Ca (mmol/L)**	**Pi (mmol/L)**	**BUN (mmol/L)**	**Scr (umol/L)**
Control group	387±49	2.43±0.06	1.22±0.24	5.60±0.48	70.11±9.60
Model group	169±20^b^	2.73±0.22^b^	4.35±1.88^b^	52.84±5.63^b^	227.48±38.21^b^
LP group	130±20^b^	2.70±0.26^b^	2.67±0.63^b,c^	52.74±3.82^b^	234.27±84.49^b^
HP group	136±17^b^	2.82±0.46^a^	2.88±0.35^b,c^	48.95±6.09^b^	178.96±31.37^b,c^
LT group	257±23^b^	2.72±0.09^b^	2.7±0.22^b,c^	27.44±4.19^b,d^	91.19±14.34^b,d^
HT group	200±22^b^	2.47±0.22^c^	2.59±0.29^b,c^	33.08±6.75^b,d^	119.53±41.95^b,d^

^a^p<0.05 versus Control group ^b^p<0.01 versus Control group

^c^p<0.05 versus Model group ^d^p<0.01 versus Model group

LP: low dose prevention, HP: high dose prevention, LT: low dose therapy, HT: high dose therapy

## DISCUSSION

Vascular calcification is a severe complication of CKD with ill-defined mechanisms. Meanwhile, there is an unmet clinical need to develop novel and efficient regiments for CKD. In this study, we analyzed the possible role of the COX-2/sclerostin axis in TGF-β1 and phosphate-induced vascular calcification in the context of CKD. We also evaluated the potential therapeutic effect of meloxicam on renal failure. Our findings demonstrate that TGF-β1 may participate in phosphate-induced vascular calcification and renal failure, probably by promoting the Wnt/β-catenin signaling through COX-2 to repress sclerostin. Our findings also suggest that the therapeutic, not the preventive, administration of COX-2-specific inhibitors may be beneficial to the treatment of renal failure.

Vascular calcification is associated with CKD, diabetes mellitus, and aging [25]. Although phosphate can induce calcification in VSMCs, the mechanism underlying this process is far more than simple precipitation of phosphate and calcium [26]. As a phosphate diet can induce vascular calcification [27], hyperphosphatemia may also be a risk factor for renal failure. In this study, we found that the serum phosphate level increased obviously in the adenine-induced renal failure rat compared to the control group. Although a high level of phosphate can induce osteoblastic trans-differentiation in vascular cells, the exact mechanism remains unclear [28]. A high level of TGF-β1 is often observed in a patient with renal failure [29]. In this study, we also found that the serum level of TGF-β1 in patients with CKD receiving no dialysis is higher than that of patients with CKD receiving regular dialysis. TGF-β1 is characteristically observed in the calcific aortic stenosis cusps and mediates the calcification of aortic valve interstitial cells through initializing apoptosis [30]. Abnormal mechanical stimulation-induced valve calcification is mediated partially through TGF-β1 [2]. Phosphate-induced calcification may implicate TGF-β1, although this idea is controversial. On the one hand, calcification of aortic segments was attenuated by the presence of TGF-β1 in *Smad3* (-/-) mice [31]. On the other hand, phosphate stimulated the expression of TGF-β1, but neutralization of TGF-β1 failed to reduce the phosphate-induced VSMCs calcification [8]. These controversial effects of TGF-β1 may due to the concentrations, cell types, and the cellular contexts. In this study, we confirmed that phosphate up regulates TGF-β1. TGF-β1 decreased the markers of smooth muscle cells, such as α-SMA and SM22α; Concurrently, TGF-β1 increased the level of calcification markers, such as Runx2 and mineralization in VSMCs. Inhibition of TGF-β signaling attenuated the phosphate-induced calcification. These data suggest that the phosphate-induced calcification of VSMCs may be partially mediated by up regulating TGF-β1. But how TGF-β1 mediates the calcification needs to be further unveiled.

Wnt/β-catenin is essential for regulating cell proliferation and differentiation, as well as the development of the skeletal system. This signaling is also associated with vascular calcification. It may promote osteogenic trans-differentiation in VSMCs through up regulating RUNX2 [3]. Ginsenoside Rb1 ameliorated the CKD-associated vascular calcification, which may be mediated by inhibiting Wnt/β-catenin through activating PPARγ [32]. High serum level of phosphate has often been described in patients with CKD [29]. High phosphate-induced osteogenic trans-differentiation in VSMCs may be mediated by activating Wnt/β-catenin signaling and down regulating Dkk-1 [33]. Here, we found that phosphate increased the level of β-catenin and p-GSK-3β in VSMCs, which supported the hypothesis that activating Wnt/β-catenin signaling may mediate the phosphate-induced calcification. Further analysis showed that phosphate increased the mRNA level of β-catenin and promoted translocation of β-catenin to the nucleus, which was reduced apparently by inhibiting TGF-β signaling. Sclerostin is an antagonist of the Wnt/β-catenin pathway through binding with LRP5/6, which is associated with high bone mass [34]. A humanized monoclonal antibody to sclerostin has been clinically tested to prevent or treat the bone fracture and/or osteoporosis through the promotion of Wnt/β-catenin signaling [35, 36]. During the early menopause, TGF-β1 may regulate bone metabolism and mediate bone turnover, and the serum level of TGF-β1 was negatively correlated with sclerostin [37]. However, TGF-β1 may also up regulate sclerostin in osteoblast to inhibit osteogenic differentiation in matured osteoblast cells [38]. Our data showed that TGF-β1 decreased the level of sclerostin in VSMCs, and sclerostin reduced the mineralization induced by TGF-β1. The different phenotypes of TGF-β1 on sclerostin may depend on the cell types, cellular contexts, concentrations, duration, and timing of administration.

Artery wall cells were induced to undergo osteogenic trans-differentiation when subjected to an inflammatory stimulus [39], which suggested that anti-inflammation might be useful to reduce calcification. COX-2 is a significant pro-inflammatory factor and can be induced by LPS, TNF-a, and hypoxia [11–14]. COX-2-specific inhibitors are used as analgesics, antipyretics, and anti-inflammatory drugs. Besides, COX-2 is also associated with bone metabolism [17, 18, 40]. Osteogenic differentiation and vascular calcification are similar physiological processes. Thus, COX-2 may also be involved in vascular calcification. In this study, we found that the serum level of COX-2 in patients with CKD without dialysis is higher than that of CKD patients receiving dialysis. Phosphate or TGF-β1 both elevated the expression of COX-2 in VSMCs, and the TGF-β1-induced calcification markers in VSMCs or vascular calcification were both decreased by COX-2 specific inhibitor (NS-398). Serum levels of COX-2 and TGF-β1 were both increased notably in the adenine-induced rat renal failure model, which implies that COX-2 may crosstalk with TGF-β1 to promote calcification. The cAMP response element (CREB) is one of the downstream effectors of COX-2. Interestingly, we found that p-Smad2/3 interacted with p-CREB and that both were recruited to the promoter of sclerostin.

Interestingly, the therapeutic administration of meloxicam was more effective than the preventive administration at mitigating the renal damage induced by adenine, as well as diminishing the serum levels of calcium, phosphate, BUN, and Src. However, other reports indicated that the use of NSAIDs as a pain killer might increase the risk of CKD, such as acetaminophen and aspirin. Still, for COX-2 specific inhibitors, a few drugs may also increase this risk of CKD, such as rofecoxib [20]. As an inducible enzyme, COX-2 was also expressed constitutively in the kidney. Besides, it was also detectable in artery endothelial and smooth muscle cells, veins, and intraglomerularly in podocytes [41]. Thus, the basal COX-2 may be primarily associated with regulating kidney perfusion and glomerular hemodynamics [41, 42]. However, if the level of COX-2 was too high because of various stimuli within the inflamed vascular system, it may promote abnormal calcification. This is why the preventive administration of meloxicam is not so effective to attenuate the renal lesion induced by adenine.

Taken together, we demonstrated that TGF-β1 might promote vascular calcification by up regulating COX-2, which then partly down regulates sclerostin. The up-regulation of sclerostin selectively in VSMCs and/or the use of COX-2-specific inhibitors may be potential therapeutic strategies to reduce renal lesion through the amelioration of vascular calcification. But the precise underlying mechanism linking TGF-β1, COX-2, and the therapeutic effects of COX-2 inhibitor on renal failure need to be further investigated.

## MATERIALS AND METHODS

### Cell culture and chemicals

Primary VSMCs were extracted from rat aorta. Cells were maintained with complete Dulbecco's modified Eagle's medium (DMEM), containing 10% fetal bovine serum, penicillin (100 U/ml), and streptomycin (100 μg/ml). Culture conditions were set at 37ºC and 5% CO_2_. Primary antibodies for TGF-β1 (sc-130348), COX-2 (sc-376861), c-Myc (sc-40), p-GSK3β (sc-373800), Runx2 (sc-390715), GAPDH (sc-47724), and β-catenin (sc-7963) were ordered from Santa Cruz Biotechnology. Primary antibodies for GSK3β (22104-1-AP), α-SMA (55135-1-AP), and sclerostin (21933-1-AP) were ordered from Proteintech (Wuhan, China). Primary antibodies for p-Smad2/3 (8828s) and p-CREB (ab32096) were ordered from CST and Abcam, respectively. NS-398 (Cat. no. N194-25MG) was ordered from Sigma-Aldrich. Meloxicam (S67664) was ordered from Yuanye Biotech (Shanghai, China).

### Primary VSMCs isolation and identification

The animal experiments were approved by the Institutional Animal Care and Use Committee (IACUC) of Chongqing Medical University (Chongqing, China). VSMCs from the rat aorta were separated, identified, and cultured according to the previous report [21]. Briefly, Sprague Dawley (SD) rats (male, 10–12 wk, 150–180 g) were ordered from and housed at the animal research center of Chongqing Medical University. The intima and adventitia were removed carefully from the aorta and then cut into small pieces and cultured with DMEM. The adherent cells were considered as VSMCs. The purity was determined by immunostaining with anti-α-SMA, a marker of VSMCs, showing that over 90% of adherent cells were VSMCs. The cells used in this study were from the 3^rd^ to 8^th^ passages.

### Construction of recombinant adenoviruses expressing TGF-β1, COX-2, sclerostin (SOST), and green fluorescent protein (GFP)

Recombinant adenovirus vectors for this study were all constructed according to the AdEasy system [22, 23]. The coding sequences of mouse *Tgf-β1*, *Cox-2*, and sclerostin were PCR amplified and subcloned into adenoviral shuttle vectors, which were then linearized for homologous recombination in BJ5183/AdEasy1 cells. The adenoviral recombinant plasmids were transduced into HEK293 (from ATCC) cells to generate recombinant adenoviruses, which were designated as AdTGF-β1, AdCOX-2, or AdSOST. A recombinant adenovirus expressing GFP only (AdGFP) was used as vehicle control.

### RNA extraction and quantitative PCR (qPCR) analysis

Total RNA from cells was extracted with TRIzol reagents (Invitrogen, USA). The template cDNAs were generated with reverse transcription (RT) kit from Takara (Cat. No. R037A). The qPCR assay was conducted using the Bio-Rad CFX Connect system with the SYBR Green mix kit (Cat. No. B21202, Bimake China). GAPDH was used as a reference gene to normalize mRNA expression levels of samples. The qPCR primers are listed in Table 2.

**Table 2:** The primers used for PCR.

**Gene**	**GenBank**	**Primer**	**Sequence (5’ →3’)**
GAPDH	X02231.1	F	AGACAGCCGCATCTTCTTGT
		R	CTTGCCGTGGGTAGAGTCAT
COX-2	NM_017232.3	F	CTCAGCCATGCAGCAAATCC
		R	GGGTGGGCTTCAGCAGTAAT
TGF-β1	NM_012775.2	F	TAGGCTGACAGCTTTGCGAA
		R	ACTGTAGCGAGAGGAGCAGA
β-catenin	NM_053357.2	F	CAGCTCCCCTGACAGAGTTG
		R	CCAGTCCGAGATCAGCAGTC
SM22a	X71070.1	F	GCCTCAACATGGCCAACAAG
		R	CAGGCTGTTCACCAACTTGC
SOST (ChIP)		Primer1 F	CAGCTGTTTTTGTCTGCCCC
		Primer1 R	GTCATCCACCTGCAGAGCTT
		Primer2 F	GCAGGAAGGCCAGAAATCCT
		Primer2 R	TAAGGGCCTGTCTCTCGGAA
		Primer3 F	AGAGGCTGGGGAGATGACAT
		Primer3 R	AACATGTCTGTGTGGGTGCA
β-catenin(ChIP)		Primer1 F	CCTGTTGCTGACATTTGCCC
		Primer1 R	ACACTCACCATCTTGCCTGG
		Primer2 F	CCTAGTCTCCAGGCCTGTCT
		Primer2 R	CTTGCTTCAGGGTCCACAGT
		Primer3 F	AGGGAGAAAGCAGCCTTTGG
		Primer3 R	GGCAAATGTCAGCAACAGGG
		F: forward; R: reverse	

### Western blot assay

Total cell lysates were harvested with radio immunoprecipitation assay (RIPA) buffer (Cat. No. R0020; Solarbio Science and Technology, China), and denatured by boiling for 10 min. A BCA assay was used to determine the protein level of each sample. Proteins were resolved with 10% SDS-PAGE and subjected to western blot assay. The presence of interest proteins was visualized by enhanced chemiluminescence (ECL) and analyzed with a Bio-Rad ChemX system (Bio-Rad, USA).

### Mineralization assay

The mineralization assay was performed as previously reported [16]. Briefly, VSMCs were cultured in mineralization medium for 20 days. At the endpoints, the cells were fixed with 0.05% glutaraldehyde for 10 min at room temperature, washed carefully with de-ionized water, and then incubated with 0.4% Alizarin Red S for five minutes, washed with de-ionized water gently and dried at room temperature. For quantitative analysis, 10% acetic acid was used to dissolve Alizarin Red S, and the absorbance was measured with a microplate reader (ELx800, BioTek Instruments, USA) at 405 nm.

### Aortic segments culture

The thoracic aorta was harvested from male SD rats and cut into 2–3 mm pieces. The aorta segments were cultured with DMEM for 24 h and then treated according to the experimental design for 14 days. At each endpoint, aorta segments were retrieved, fixed, and embedded with paraffin for further histological and histochemical evaluation.

### Von Kossa staining

After being deparaffined, aortic sections were submerged in AgNO_3_ solution (2%) and treated with ultraviolet light for 20 to 60 min simultaneously, followed by incubation with sodium thiosulphate for 2 min after being washed carefully with de-ionized water twice. The sections were stained with hematoxylin and eosin, dehydrated, cleared and mounted as conventional methods.

### Immunofluorescent staining

VSMCs were seeded to 48-well culture plates and treated according to the experimental design. At each endpoint, cells were fixed with formaldehyde (4%) and rinsed gently with PBS (4ºC), permeabilized with 0.1% Triton X-100 for 10 min, and washed with PBS (4ºC) immediately, followed by treatment with H_2_O_2_ for 10 min and washing with PBS. The cells were then incubated with an antibody to β-catenin overnight (4ºC) or isotype IgG of the mouse, and subsequently anti-mouse IgG488 (A23210-1, Abbkine China Branch). Finally, cells were stained with DAPI (1 μg/ml) and washed extensively with PBS (4ºC). Images were recorded with a fluorescence microscope.

### Enzyme-linked immunosorbent assay (ELISA)

The ELISA kits were ordered from Elabscience Biotechnology Co. Ltd. (human TGF-β1, Cat. No. E-EL-H0110c; human COX-2, Cat. No. E-EL-H1846c; rat TGF-β1, Cat. No. E-EL-R0084c; rat COX-2, Cat. No. E-EL-R0792c). The clinical and rat samples were used for this assay. The immunoassay was conducted according to the manufacturer's instructions. The absorbance was measured with a microplate reader at 450 nm (ELx800; BioTek Instruments, Inc.).

### Transfection and luciferase reporter assay

The reporter plasmids, pBGluc-Smad and pBGluc-TCF/LEF, were kindly provided by Dr. T.-C. He of The University of Chicago Medical Center. Briefly, cells were seeded in T25 cell culture flasks and transfected with 2 μg of reporter plasmids per flask with Lipofectamine (Invitrogen). At 16 h after transfection, the cells were trypsinized and re-seeded into 24-well cell culture plates and treated with different concentrations of phosphate (0, 1.5, 2.5, and 3.5 mM). After 48 h, the cell culture medium was taken for Gaussia Luciferase assays using the NEB kit (Cat. No. E3300s). The activities of Gaussia Luciferase were normalized with protein levels of samples, which were determined by the BCA assay.

### Animal model of renal failure

Renal failure was established in rats as described [24]. Sixty SD rats (male, 10–12 w, 150–200 g) were bought from the animal research center of the Chongqing Medical University. The animal experiments for this study were approved by the IACUC of the Chongqing Medical University (Chongqing, China). Rats were randomly divided into six groups, including control group (normal diet 6 w), low-dose prevention group (0.75% adenine diet and 1 mg/kg meloxicam 6 w), high-dose prevention group (0.75% adenine diet and 6 mg/kg meloxicam 6 w), model group (0.75% adenine diet 6w), low-dose therapy group (after treating with 0.75% adenine diet 6 w, intragastrically administrating 1 mg/kg meloxicam for 20 d), and high-dose therapy group (after treating with 0.75% adenine diet 6 w, intragastrically administrating 6 mg/kg meloxicam for 20 d).

At each endpoint, the rats were sacrificed, and serum and renal tissues were collected and stored at −80ºC or fixed with formaldehyde. Thoracic arteries were also harvested for Von Kossa staining and assessment of the protein levels of TGFβ1 and COX-2.

### Clinical samples

The use of patient samples in this study was approved by the Medical Ethics Committee of The First Affiliated Hospital of Chongqing Medical University. The signed informed consent was obtained from each patient. Serum samples (n=40 or 48) were obtained from a subset of patients with CKD at the time of diagnosis.

### Immunoprecipitation (IP) assay

After treatment for 30 h, cells were washed with PBS (4°C) and lysed with RIPA buffer (Cat. No. R0020, Solarbio Science and Technology, China) on ice, which was supplemented with an inhibitor cocktail for proteases and phosphatases. Antibody for p-Smad2/3 or p-CREB was used for IP. The precipitants were boiled for 10 min with RIPA buffer, resolved with 10% SDS-PAGE, and subjected to western blot analysis.

### Chromatin immunoprecipitation (ChIP) assay

After treatment with AdTGF-β1 for 30 h, cells were cross-linked and subjected to ChIP analysis as previously described [16]. Specific antibodies for p-Smad2/3, p-CREB, or goat IgG were used to pull down the protein-DNA complexes. Enrichment of promoter fragment for sclerostin or β-catenin was detected by PCR, and PCR primer sequences are listed in Table 2.

### Statistical analysis

For statistical analysis, all data were presented as means ± SDs. Data were gathered from at least three independent experiments. Statistical analysis was performed with Prism software (version 6, GraphPad Software Inc., La Jolla, CA, USA). The difference between the two groups was analyzed with a two-tailed Student's *t*-test, and the difference among multiple groups was analyzed with a one-way analysis of variance (ANOVA) with Tukey's post hoc test. The difference was considered significant if the *p*-value was less than 0.05.
